# Phenotypic and Genotypic Characterization of a Hypervirulent Carbapenem-Resistant Klebsiella pneumoniae ST17-KL38 Clinical Isolate Harboring the Carbapenemase IMP-4

**DOI:** 10.1128/spectrum.02134-21

**Published:** 2022-02-28

**Authors:** Jintao He, Xiaoxing Du, Xi Zeng, Robert A. Moran, Willem van Schaik, Quanming Zou, Yunsong Yu, Jinyong Zhang, Xiaoting Hua

**Affiliations:** a Department of Infectious Diseases, Sir Run Run Shaw Hospital, Zhejiang University School of Medicine, Hangzhou, Zhejiang, China; b Key Laboratory of Microbial Technology and Bioinformatics of Zhejiang Province, Hangzhou, Zhejiang, China; c Regional Medical Center for National Institute of Respiratory Diseases, Sir Run Run Shaw Hospital, School of Medicine, Zhejiang University, Hangzhou, Zhejiang, China; d National Engineering Research Center of Immunological Products, Department of Microbiology and Biochemical Pharmacy, College of Pharmacy, Third Military Medical University, Chongqing, People’s Republic of China; e Department of Pharmacy, The 78th Group Army Hospital of Chinese PLA, Mudanjiang, Heilongjiang, China; f Institute of Microbiology and Infection, College of Medical and Dental Sciences, University of Birmingham, Birmingham, United Kingdom; Peking University People’s Hospital

**Keywords:** *Klebsiella pneumoniae*, ST17, *bla*_IMP-4_, carbapenem resistant, hypervirulence

## Abstract

Carbapenem-resistant hypervirulent Klebsiella pneumoniae (CR-hvKP) is a threat to global public health. We characterized a sequence type 17 (ST17) K. pneumoniae clinical isolate that was resistant to carbapenems and belonged to serotype KL38/O2. Its complete genome is comprised of a 5.1-Mb chromosome and two conjugative plasmids. The 52,578-bp N-type plasmid pXH210-IMP contains the *bla*_IMP-4_ carbapenemase gene and the quinolone resistance gene *qnrS1*. The 272,742-bp FII(K)-9:FIB(K)-10 plasmid pXH210-AMV carries an array of genes that confer resistance to aminoglycosides, chloramphenicol, quinolones, tetracycline, sulfonamides, trimethoprim, arsenic, copper, and silver. However, the XH210 genome otherwise lacks the genes that are considered characteristic markers of hypervirulence in K. pneumoniae. The virulence potential of XH210 was assessed using a random forest algorithm predictive model, as well as Galleria mellonella and mouse infection models. The results of these were concordant and suggested that XH210 is hypervirulent and therefore a CR-hvKP strain. This worrying convergence of virulence and clinically significant antibiotic resistance is particularly concerning given the absence of typical hypervirulence markers. Further investigations are required to understand the virulence mechanisms of XH210 and to improve the diagnostics of hypervirulent K. pneumoniae.

**IMPORTANCE** The combination of drug resistance and hypervirulence significantly limits the available treatment options for life-threatening infections caused by multidrug-resistant hvKP, especially CR-hvKP. To date, research on IMP-producing CR-hvKP is extremely scarce, and the virulence mechanisms of CR-hvKP are far more complicated and diverse than has been described in the literature so far. In this study, we characterized the tigecycline-resistant and IMP-4 carbapenemase-producing ST17 K. pneumoniae isolate XH210 from a human blood sample. Importantly, XH210 exhibits hypervirulence but does not possess traits that are frequently associated with the phenotype, highlighting the urgent need to improve identification of potentially hypervirulent isolates and enhance active surveillance of CR-hvKP strains to prevent their dissemination.

## INTRODUCTION

Klebsiella pneumoniae is a well-studied Gram-negative bacterium of the *Enterobacterales* family and a prominent cause of community-acquired and nosocomial infections (1–3). Globally distributed antimicrobial-resistant clones, particularly carbapenem-resistant K. pneumoniae (CRKP), pose serious therapeutic challenges (4). Among the K. pneumoniae population, hypervirulent K. pneumoniae (hvKP) strains exhibit increased virulence relative to classical K. pneumoniae (cKP) strains. Worrisomely, hvKP is becoming increasingly resistant to antibiotics through the acquisition of multiple antibiotic resistance genes, while drug-resistant cKP strains can acquire virulence genes and increase their virulence potential (5).

XH209 is a CRKP that was isolated in 2014 from the blood of a patient in Hangzhou, China, at the outset of tigecycline treatment (6). Initially, it was mistakenly believed that XH209 did not contain a carbapenemase gene or carry plasmids (7). A second isolate of the same strain, XH210, was isolated from the same patient after tigecycline treatment and shown to contain a mutation associated with tigecycline resistance (8) but has not been further characterized.

Here, we determined the complete genome sequence of K. pneumoniae XH210 using short- and long-read sequence data. The complete XH210 genome was characterized to assess both its antibiotic resistance and virulence potential. The transferability of multidrug resistance plasmids in XH210 was determined in the laboratory. The virulence of XH210 was assessed using the random forest algorithm predictive model and confirmed via mouse pneumonia and Galleria mellonella infection models.

## RESULTS

### Isolate characteristics.

The genome of K. pneumoniae XH210 consists of a 5,120,204-bp chromosome and two plasmids (Table 1). XH210 belongs to sequence type 17 (ST17), its capsule type is KL38, and its O antigen type is O2. Antimicrobial susceptibility testing (AST) revealed that XH210 exhibits resistance to most tested antibiotics but is susceptible to amikacin (Table 2). The XH210 genome contains multiple antibiotic resistance genes, including ones that confer resistance to aminoglycosides (*aacC2d* and *strAB*), β-lactams (*bla*_SHV-94_, *bla*_SHV-96_, *bla*_SHV-172_, *bla*_IMP-4_, and *bla*_CTX-M-14_), fosfomycin (*fosA*), quinolones (*oqxAB* and *qnrS1*), phenicols (*catA2*), sulfonamides (*sul1* and *sul2*), tetracycline [*tet*(A) and *tet*(D)], and trimethoprim (*dfrA1*). *bla*_SHV-94_, *bla*_SHV-96_, *bla*_SHV-172_, *oqxAB*, and *fosA* are located in the XH210 chromosome. The smaller plasmid, pXH210-IMP, carries both the *qnrS1* and *bla*_IMP-4_ genes, while the remaining resistance genes are located in the larger plasmid, pXH210-AMV.

**TABLE 1:** Genomic characteristics of K. pneumoniae isolate XH210

Genetic material	Replicon type	Size (bp)	GC content (%)	Antimicrobial resistance gene(s)			
				β-Lactam(s)	Aminoglycoside	Fluoroquinolone	Others
Chromosome		5,120,204	57.6	*bla*_SHV-94_, *bla*_SHV-96_, *bla*_SHV-172_		*oqxAB*	*fosA*
pXH210-IMP	N	52,578	51.1	*bla*_IMP-4_		*qnrS1*	
pXH210-AMV	FII(K)-9, FIB(K)-10	272,742	51.8	*bl*a_CTX-M-14_	*aacC2d*, *strAB*	*qnrS1*	*catA2*, *sul1*, *sul2*, *tet*(D), *tet*(A), *dfrA1*

**TABLE 2:** Antimicrobial susceptibilities of K. pneumoniae XH210, recipient strains, and transconjugants

Strain	Annotation	MIC (μg/mL) of^*a*^:							
		MEM	IPM	CHL	TET	AK	CTX	FEP	CRO
XH210	The original isolate	16	4	>256	>256	1	128	128	>128
XH1538	J53 transconjugant with pXH210-IMP	4	2	4	0.5	2	256	128	>128
XH1539	J53 transconjugant with pXH210-AMV	≤0.0625	0.25	>256	256	4	16	64	>128
XH1540	XH1541 transconjugant with pXH210-IMP and pXH210-AMV	8	4	>256	>256	0.5	32	32	64
XH1541	K. pneumoniae ATCC 13883-Rif^r^, rifampicin resistant	≤0.0625	0.5	4	1	0.5	≤0.25	≤0.125	≤0.125
J53	E. coli J53, sodium azide resistant	≤0.0625	0.125	4	1	2	≤0.25	≤0.125	≤0.125

a MEM, meropenem; IPM, imipenem; CHL, chloramphenicol; TET, tetracycline; AK, amikacin; CTX, cefotaxime; FEP, cefepime; CRO, ceftriaxone.

**FIG 1:**
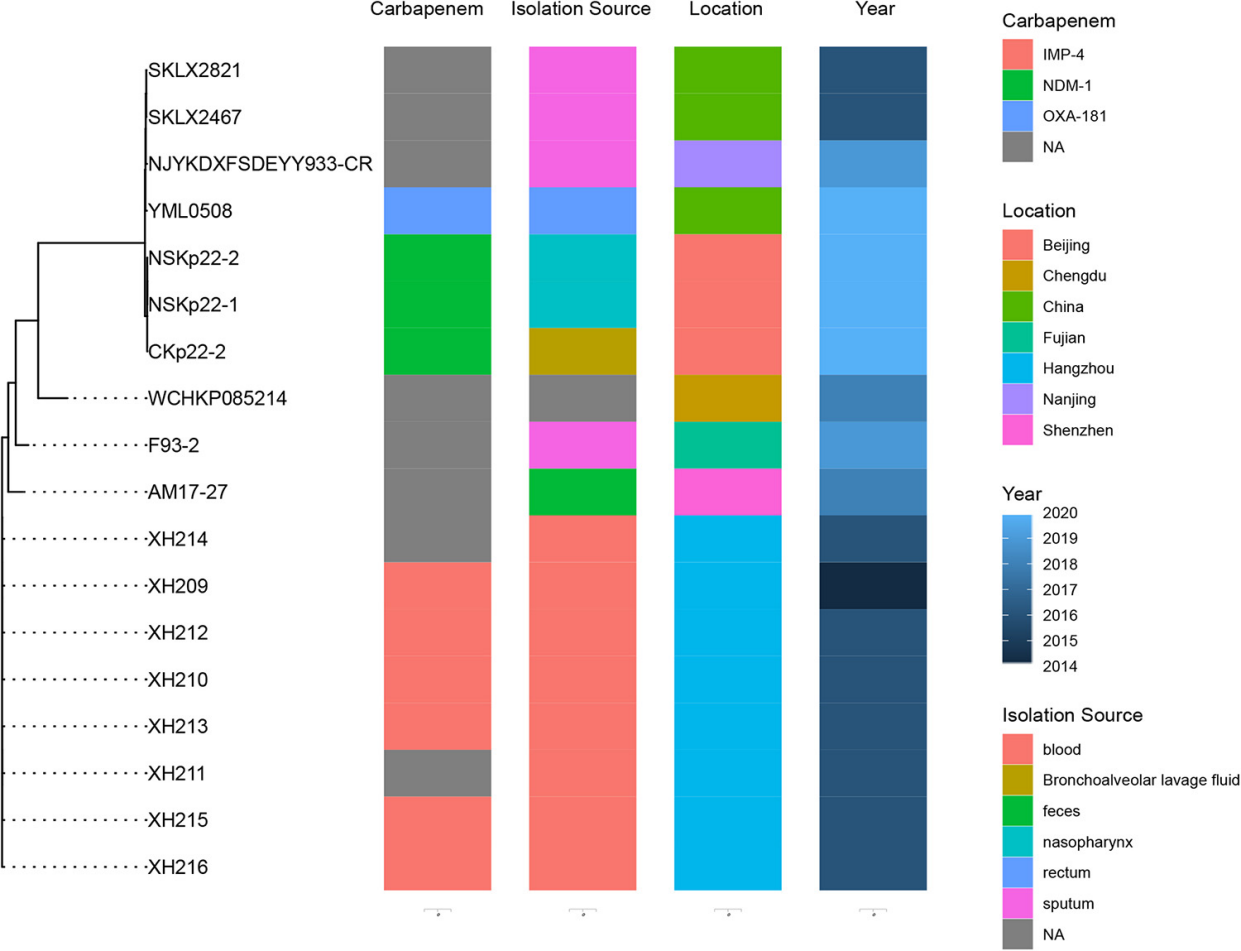
The phylogenetic tree of ST17 isolates. Eighteen ST17 K. pneumoniae genome sequences were used. Information for the ST17 strains is shown on the right, including carbapenemase gene, isolation source, location, and time.

We constructed a core genome phylogenetic tree using XH210 and 17 further ST17 K. pneumoniae strains isolated in China, which divided into two clusters (Fig. 1). The first cluster is comprised of isolates derived from North China, some carrying carbapenemase gene *bla*_NDM-1_ or *bla*_OXA-181_. The second cluster contains isolates from West China and South China, some of which, including XH210, harbor carbapenemase gene *bla*_IMP-4_.

### Characterization of pXH210-AMV and pXH210-IMP.

pXH210-AMV is a 272,742-bp F-type plasmid that contains FII(K)-9 and FIB(K)-10 replicons. Although it has been heavily modified by translocatable elements, pXH210-AMV contains a complete and uninterrupted F-like transfer region (9). The antibiotic resistance genes in pXH210-AMV confer resistance to multiple classes of antibiotics (Table 1), and the plasmid also contains genes expected to confer resistance to silver (*silABCERS*), copper (*pcoABCDRS*), and arsenic (*ars*).

**FIG 2:**
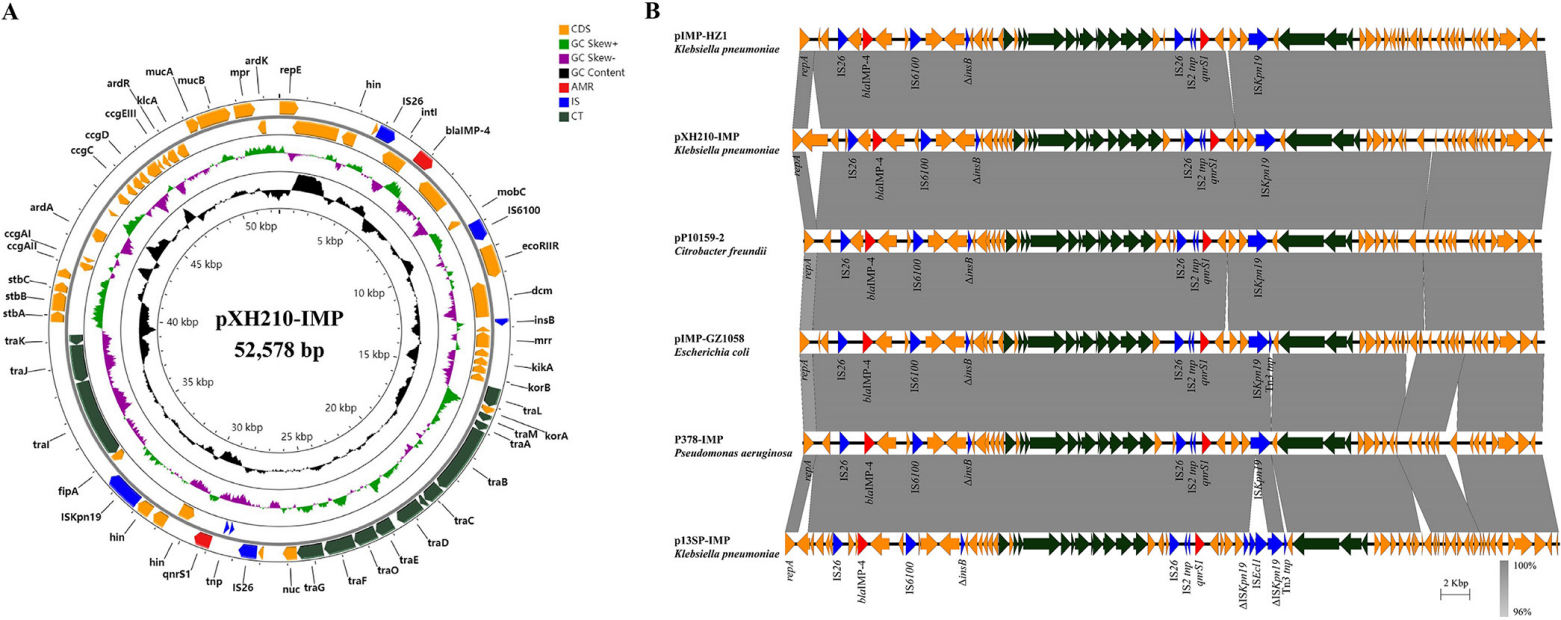
Characterization of *bla*_IMP-4_-carrying plasmid pXH210-IMP. (A) Circular map of pXH210-IMP. AMR, antimicrobial resistance genes; IS, insertion sequence-associated genes; CT, conjugal transfer-related genes. (B) Scaled, linear sequence comparison between plasmids pXH210-IMP, pIMP-HZ1 (KU886034), pP10159-2 (MF072962), pIMP-GZ1058 (KU051709), P378-IMP (KX711879), and p13SP-IMP (MH909334). Antibiotic resistance genes are shown in red arrows. The individual conjugation-related genes are shown with dark green arrows. Blue arrows show insertion sequence-associated genes. The other genes are shown as orange arrows.

pXH210-IMP is an N-type plasmid that contains *bla*_IMP-4_ and *qnrS1* in two different insertion regions (Fig. 2A). The *bla*_IMP-4_ gene is located in a group II intron-containing class 1 integron that has previously been designated In*823*::*Kl.pn.I3* when found in pIMP-HZ1 (GenBank accession number KU886034), the first sequenced *bla*_IMP-4_-carrying plasmid (10). The *qnrS1* genes in pXH210-IMP and pIMP-HZ1 are found in 2,747-bp and 2,959-bp segments, respectively, between copies of IS*26* and IS*Kpn19*. An IS*Kpn19*-mediated deletion is responsible for the shorter segment in pXH210-IMP. The backbones of pXH210-IMP and pIMP-HZ1 differ in the lengths of two short repeat regions associated with the equivalent to the resolvase gene of R46 and with the origin of transfer (*oriT*). pXH210-IMP contains a complete and uninterrupted R46-like transfer region that contains all determinants required for conjugative transfer. A number of plasmids from different bacterial hosts are closely related to pXH210-IMP and pIMP-HZ1 (Fig. 2B), indicating that this lineage of *bla*_IMP-4_-harboring N-type plasmids is widely disseminated.

### Both antibiotic resistance plasmids in XH210 are conjugative.

**FIG 3:**
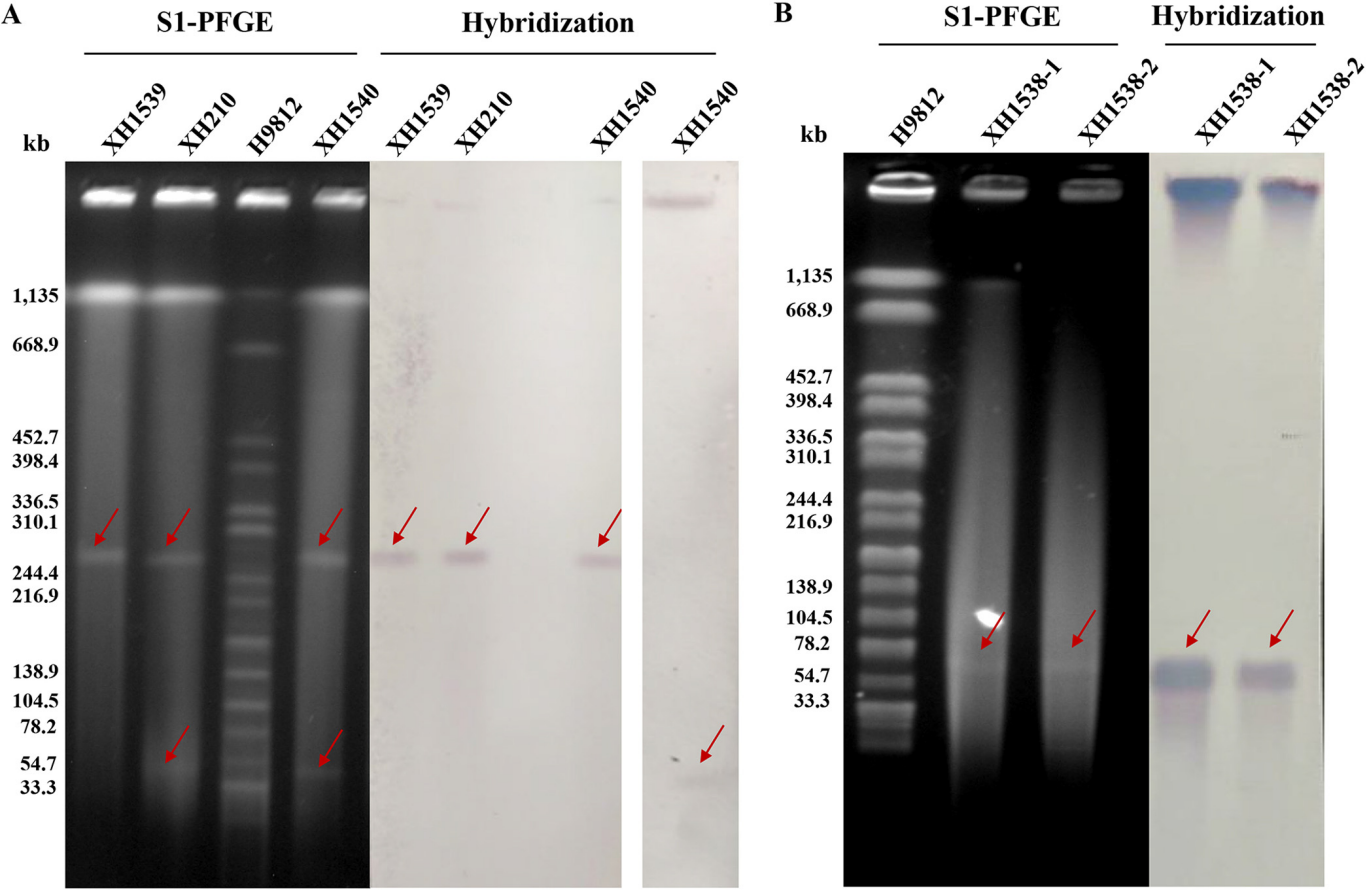
S1 nuclease-digested plasmid DNA and Southern blot hybridization of XH210 and corresponding transconjugants XH210, XH1539, and XH1540 (A) and of XH1538 (B). The red arrows show positive signals via Southern blot hybridization with a *bla*_IMP-4_-specific (for pXH210-IMP) or *catA2*-specific (for pXH210-AMV) probe.

To test the transferability of pXH210-IMP and pXH210-AMV, we performed conjugation experiments where XH210 was the donor and Escherichia coli strain J53 or K. pneumoniae strain XH1541 (ATCC 13883-Rif^r^) was the recipient. pXH210-IMP transferred to strains J53 and XH1541 at mean frequencies of 4.7 × 10^−5^ and 1.3 × 10^−4^ transconjugants per donor cell (TC/D), respectively, while pXH210-AMV transferred to J53 and XH1541 at 9.9 × 10^−7^ and 9.7 × 10^−7^ TC/D, respectively. According to S1 nuclease pulsed-field gel electrophoresis (S1-PFGE) and the corresponding Southern blot hybridizations, XH210 contains two plasmids with sizes of ∼54 kb and ∼270 kb, and *bla*_IMP-4_ is located in the ∼54-kb plasmid, consistent with the sequence data (Fig. 3). These two plasmids were transferred separately into E. coli J53, while they were cotransferred into K. pneumoniae XH1541 using the same methods. AST showed that the K. pneumoniae transconjugant XH1540 exhibited resistant phenotypes similar to those of XH210, although the MICs of some agents were lower than those of the donor (Table 2). E. coli transconjugant XH1538, carrying pXH210-IMP, exhibited resistance to all tested β-lactams, including cefotaxime, cefepime, ceftriaxone, imipenem, and meropenem, but remained susceptible to chloramphenicol and tetracycline. In contrast, E. coli transconjugant XH1539, carrying pXH210-AMV, exhibited resistance to chloramphenicol and tetracycline but remained susceptible to imipenem and meropenem. This indicated that pXH210-IMP accounts for the carbapenem resistance phenotype of XH210, while pXH210-AMV accounts for the chloramphenicol and tetracycline resistance phenotypes, as predicted from the sequence data.

### Comparing the virulence of K. pneumoniae strains ATCC 700721, NTUH-K2044, and XH210 in G. mellonella larvae and the mouse pneumonia model.

**FIG 4:**
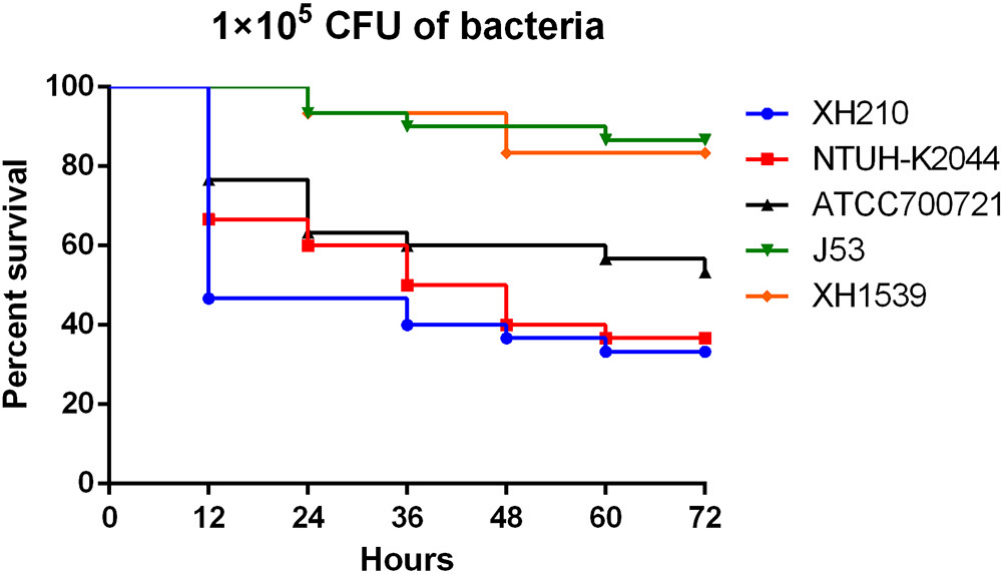
Virulence of individual isolates in the G. mellonella model. Larvae were inoculated with 10^5^ CFU of XH210, NTUH-K2044, or PBS. Survival was monitored every 12 h for 3 days. The experiment was repeated in biological triplicate, and the data are the mean values.

**FIG 5:**
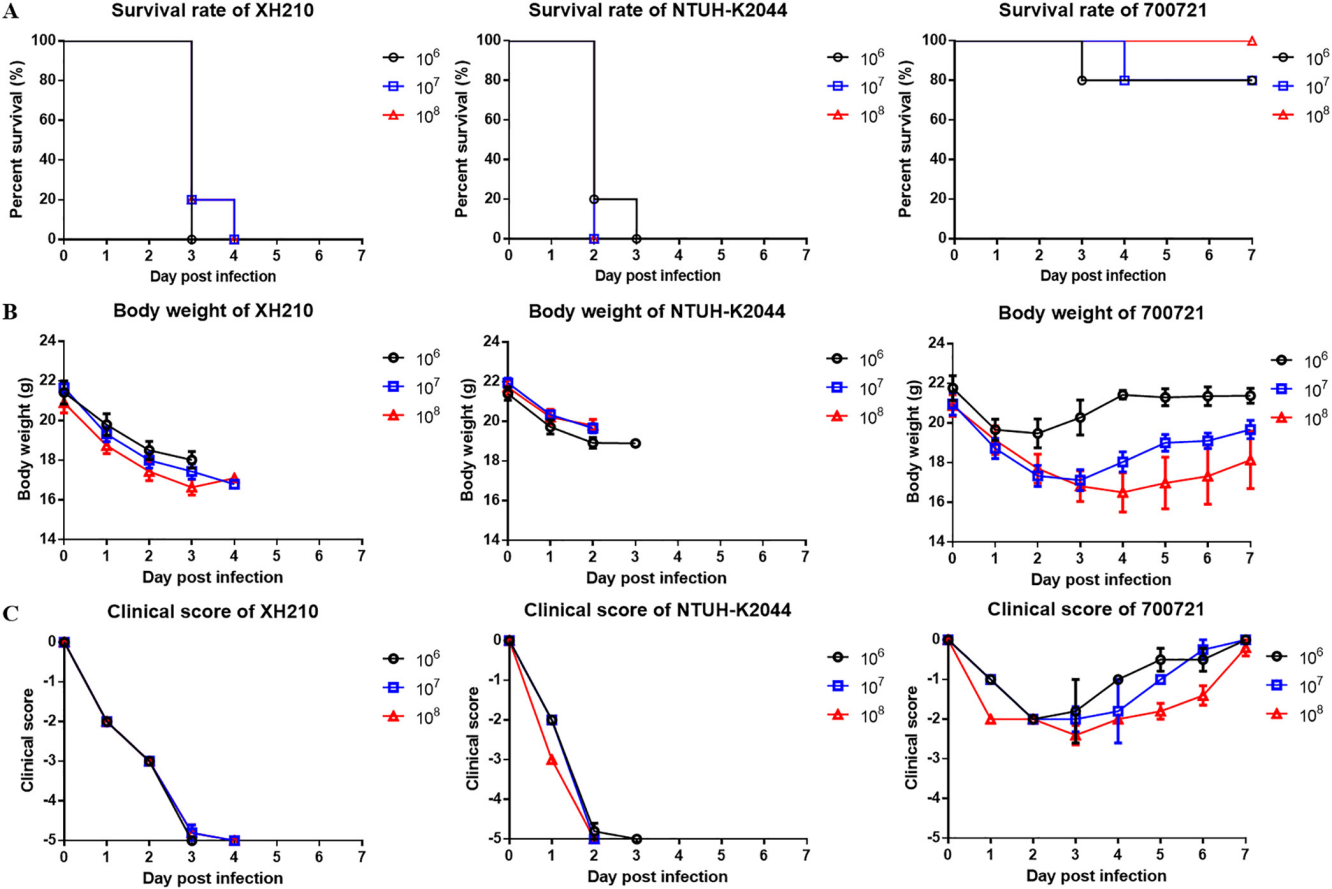
Virulence of ATCC 700721, NTUH-K2044, and XH210 in mouse pneumonia model. Mice were infected with 10^6^, 10^7^, or 10^8^ CFU of K. pneumoniae intratracheally, and the survival rates (A), body weights (B), and clinical scores (C) of mice were recorded for 7 days.

Allelic profiles of XH210 core genes were input into the virulence prediction model of Lan et al. (11), which predicted that the isolate was hypervirulent. To confirm this, we investigated the susceptibility of G. mellonella larvae to K. pneumoniae XH210 and strain NTUH-K2044, which is used as a hypervirulent reference strain in molecular pathogenesis studies (12). K. pneumoniae ATCC 700721 was used as a nonhypervirulent control strain. The results showed that when larvae were injected with 1 × 10^5^ CFU of one of the strains, identical trends were observed between the XH210 and ATCC 700721 infections, with no statistical difference (*P* = 0.087). The mortality of larvae was similar or even slightly higher for XH210 than for NTUH-K2044 (Fig. 4), indicating that XH210 exhibited a virulence level similar to that of the hvKP control strain. A mouse pneumonia model was employed to further evaluate the *in vivo* virulence of XH210. BALB/c mice were intratracheally infected with 1 × 10^6^, 1 × 10^7^, or 1 × 10^8^ CFU of K. pneumoniae, and the survival rates, body weights, and clinical scores of the mice were recorded. All mice infected with XH210 or NTUH-K2044 succumbed to the infection, even when the infective dose was reduced to 1 × 10^6^ CFU. In contrast, up to 80% of mice inoculated with 1 × 10^8^ CFU of ATCC 700721 survived the infection (Fig. 5A). The weight loss of mice infected with ATCC 700721 was dependent on the inoculated dose (Fig. 5B), and the clinical scores of mice that received ATCC 700721 generally recovered by day 7 (Fig. 5C). In contrast, the body weights and clinical scores of mice infected with XH210 or NTUH-K2044 declined continuously until death (Fig. 5B and C). These results suggest that, like NTUH-K2044, XH210 is a hypervirulent strain, supporting the random forest model’s prediction and the results from the G. mellonella model.

## DISCUSSION

The emergence of hvKP that can cause severe infections with high mortality rates in apparently healthy people has raised serious concern globally (13). HvKP strains are usually susceptible to most antimicrobials apart from ampicillin, but like cKP lineages, hvKP strains have acquired resistance to multiple antimicrobial agents in recent years (14). Meanwhile, drug-resistant cKP strains have obtained hvKP-specific virulence factors (5). It has been predicted that drug-resistant cKP strains are more likely to acquire virulence genes than hvKP strains are to acquire antibiotic resistance genes (15). The combination of drug resistance and hypervirulence significantly limits the available treatment options for life-threatening infections caused by multidrug-resistant hvKP, especially carbapenem-resistant hvKP (CR-hvKP). In this study, we characterized K. pneumoniae isolate XH210, an ST17 CR-hvKP isolate from a human blood sample.

Although it is difficult for clinical laboratories to distinguish hvKP and cKP, serotyping and genomic background characterization can provide valuable information regarding the definition of hvKP. To date, the K1 and K2 capsular serotypes have accounted for approximately 70% of hvKp isolates (5, 7). Detection of five genotypic markers for a virulence plasmid (*peg-344*, *iroB*, *iucA*, *rmpA*, and *rmpA2*) coupled with detection of siderophore production showed >0.95 diagnostic accuracy for differentiating hvKP from cKP (16). However, a contrary report suggested that virulence factors (*rmpA*, *iucA*, positive string test, and pLVPK) were poor predictors for hvKP (5). A random forest model based on the core genome allelic profile showed >0.98 diagnostic accuracy for hvKP (11). G. mellonella larvae have been considered a consolidated *in vivo* infection model for K. pneumoniae (17, 18). However, Russo and MacDonald reported that while a murine infection model accurately differentiated hvKP from cKP, a G. mellonella model did not (19). In this study, K. pneumoniae XH210 was predicted to be hypervirulent using the random forest algorithm predictive model. We used G. mellonella and murine models to confirm the virulence of K. pneumoniae XH210, which belonged to KL38/O2 and did not contain any of the significant virulence factors described above. The most obvious putative virulence genes in pXH210-AMV appear to be *fecABCDER*, which encode a siderophore system for iron acquisition. However, no statistical difference was observed between J53 and XH1539 (J53 harboring pXH210-AMV) in virulence experiments (Fig. 4). We hypothesize that the hypervirulence of XH210 is determined by a variety of factors, such as a type VI secretion system (TssBCJFG), fimbrial proteins (MrkABCDFHIJ), capsule biosynthesis proteins (RcsA and RcsB), and possibly FecABCDER, but the exact mechanisms remain unclear. Our results also confirmed the correlation between G. mellonella and murine infection models for virulence, but strong conclusions cannot be drawn due to the limited numbers of strains tested here. Further investigations are required to strengthen our understanding of the virulence mechanisms of XH210.

We demonstrated that both pXH210-AMV and pXH210-IMP were conjugative plasmids that could be transferred from XH210 to E. coli and K. pneumoniae recipients. pXH210-IMP is closely related to pIMP-HZ1, from a K. pneumoniae isolate obtained from a patient from Huizhou in 2010 (10). Both pXH210-IMP and pIMP-HZ1 could be transferred by conjugation to an E. coli recipient at high frequencies, but the efficiency of pXH210-IMP (4.7 × 10^−5^ per donor cell) was slightly lower than that of pIMP-HZ1 (1.2 × 10^−4^ per donor cell). This observation of a conjugative carbapenem resistance plasmid in hvKP XH210 is important, as it has been postulated that hvKP strains are less likely to acquire carbapenemase plasmids than cKP strains (15).

In conclusion, the CR-hvKP clinical isolate XH210 was characterized as ST17 KL38/O2 serotype and was hypervirulent, which was confirmed by the random forest algorithm predictive model, G. mellonella larva infection model, and mouse pneumonia model. XH210 exhibited resistance to most tested antibiotics but was susceptible to amikacin. Most of XH210’s resistance determinants were found in two conjugative plasmids, a widely disseminated *bla*_IMP-4_-carrying N-type plasmid and a large F-type plasmid carrying antibiotic and metal resistance genes. Importantly, XH210 exhibited hypervirulence but did not possess traits that are frequently associated with the phenotype. This suggests that further work is required to improve the identification of potentially hypervirulent isolates and that active surveillance of CR-hvKP strains should be implemented urgently.

## MATERIALS AND METHODS

### Whole-genome sequencing and sequence analysis.

The genomic DNA of XH210 was extracted using the QIAamp DNA minikit (Qiagen, Valencia, CA) and sequenced on the HiSeq X ten (Illumina, San Diego, CA, USA) and MinION platforms (Nanopore, Oxford, UK) at Zhejiang Tianke (Hangzhou, China). The Illumina and Nanopore reads were hybrid assembled using Unicycler version 0.4.8 (20). Assembled contigs were annotated using Prokka (21). Resfinder was used to identify antimicrobial resistance genes (https://cge.cbs.dtu.dk/services/ResFinder/). The PlasmidFinder, pMLST, and KpVR tools were used to detect and type plasmid replicons (22). The virulence prediction model was built using a Random Forest algorithm based on core genome allelic profiles of K. pneumoniae strains (11).

### Phylogenetic analysis.

A collection of K. pneumoniae ST17 genomes were obtained from NCBI (Table 3). The core genome phylogeny was constructed using Roary version 3.12.0 (23). A phylogenetic tree was generated with FastTree version 2.1.10 (24), and the output graphic file was generated via ggtree (25).

**TABLE 3:** ST17 K. pneumoniae genome sequences downloaded from NCBI for comparison to XH210

Strain	Assembly accession no.	Yr	Location	Host	Isolation source	Carbapenemase
NSKp22-1	GCA_011683205.1	2020	Beijing	Homo sapiens	Nasopharynx	NDM-1
NSKp22-2	GCA_011683185.1	2020	Beijing	Homo sapiens	Nasopharynx	NDM-1
CKp22-2	GCA_011683155.1	2020	Beijing	Homo sapiens	Bronchoalveolar lavage fluid	NDM-1
YML0508	GCA_009884395.1	2020	China	Homo sapiens	Rectum	OXA-181
NJYKDXFSD EYY933-CR	GCA_006130295.1	2019	Nanjing	Homo sapiens	Sputum	NA^*a*^
F93-2	GCA_004120175.1	2019	Fujian	Homo sapiens	Sputum	NA
AM17-27	GCA_003471715.1	2018	Shenzhen	Homo sapiens	Feces	NA
WCHKP085214	GCA_003037795.1	2018	Chengdu	Homo sapiens	NA	NA
SKLX2821	GCA_001701615.1	2016	China	Homo sapiens	Sputum	NA
SKLX2467	GCA_001701585.1	2016	China	Homo sapiens	Sputum	NA
XH210	GCA_001699105.1	2016	Hangzhou	Homo sapiens	Blood	IMP-4
XH216	GCA_001699095.1	2016	Hangzhou	Homo sapiens	Blood	IMP-4
XH215	GCA_001699045.1	2016	Hangzhou	Homo sapiens	Blood	IMP-4
XH214	GCA_001699035.1	2016	Hangzhou	Homo sapiens	Blood	NA
XH213	GCA_001699025.1	2016	Hangzhou	Homo sapiens	Blood	IMP-4
XH211	GCA_001699015.1	2016	Hangzhou	Homo sapiens	Blood	NA
XH212	GCA_001698945.1	2016	Hangzhou	Homo sapiens	Blood	IMP-4
XH209	GCA_000775955.1	2014	Hangzhou	Homo sapiens	Blood	IMP-4

a NA, not available.

### Plasmid transfer experiments.

Conjugation experiments were carried out by filter mating using rifampicin-resistant K. pneumoniae strain XH1541 and the sodium azide-resistant E. coli strain J53 as the recipients, as described previously (26). Transconjugants were selected using MH agar plates containing 1 μg/mL meropenem and 300 μg/mL sodium azide (for pXH210-IMP), 1 μg/mL meropenem and 300 μg/mL rifampicin (for pXH210-IMP), 100 μg/mL chloramphenicol and 300 μg/mL sodium azide (for pXH210-AMV), or 100 μg/mL chloramphenicol and 300 μg/mL rifampicin (for pXH210-AMV). Conjugation frequencies were calculated by dividing the number of transconjugants (CFU/mL) by the number of donor cells (CFU/mL). PCR analysis and MIC profiling were carried out to determine the difference between the parental strain and the corresponding transconjugants. S1-PFGE and Southern blotting were performed as described previously (27). DNA fragments were hybridized with a digoxigenin-labeled *bla*_IMP-4_-specific or *catA2*-specific probe. Salmonella enterica serotype Braenderup H9812 digested with XbaI was used as a size marker.

### Antimicrobial susceptibility testing.

The original strain XH210, the recipient strains E. coli J53 and XH1541, and transconjugants were tested for their susceptibility to imipenem, meropenem, chloramphenicol, tetracycline, amikacin, cefotaxime, cefepime, and ceftriaxone by the broth microdilution method according to the guidelines provided by the Clinical and Laboratory Standards Institute (28). The E. coli strain ATCC 25922 was used for quality control.

### Galleria mellonella infection model.

The survival of G. mellonella larvae was assayed as previously described (29). Log-phase cell cultures were centrifuged and resuspended in phosphate-buffered saline (PBS) to 10^7^ CFU/mL. Ten larvae were injected with 10 μL of bacterial suspension and incubated at 37°C in darkness. Ten microliters of PBS was injected in parallel as a control group. Viability was assessed by checking for movement every 12 h, and the dead larvae were counted for 3 days. The experiment was repeated in biological triplicate.

### Virulence evaluation in mouse pneumonia model.

Six- to 8-week-old female BALB/c mice that weighed 18 to 22 g were purchased from Hunan SLAC Jingda Laboratory Animal Co. Ltd. (Hunan, China) and kept under specific-pathogen-free conditions. All mouse experiments were approved by the Animal Ethical and Experimental Committee of Third Military Medical University. Five mice were included in each group. Intraperitoneal injection of pentobarbital sodium (75 mg/kg of body weight) was used to anesthetize the mice, and K. pneumoniae diluted in 20 μL PBS was inoculated intratracheally (30). The actual concentrations of inoculated bacteria were determined by plating serial dilutions on LB agar plates. The survival rates, body weights, and clinical scores of mice were continuously observed and recorded for 7 days after infection. The evaluation standard for clinical scores was assessed as described previously (31). The experiment was repeated in biological triplicate. The log-rank test for survival rate and Student’s *t* test for body weight and clinical score were performed using GraphPad Prism 6.

### Data availability.

The complete genome sequences of the K. pneumoniae XH210 isolate were deposited in GenBank under accession numbers CP052761 to CP052763.
